# Fire safety status and evacuation of medical facility considering elevated oxygen concentrations

**DOI:** 10.1016/j.heliyon.2024.e36847

**Published:** 2024-08-31

**Authors:** Mohsin Ali Shaikh, Rehmat Karim, Nashiru Mumuni Daniel, Mujeeb Ali Khan

**Affiliations:** State Key Laboratory of Fire Science, University of Science and Technology of China, JinZhai Road 96, Hefei, Anhui, 230026, China

**Keywords:** Hospital, Evacuation, Pathfinder, Fire dynamic simulator, Required safe evacuation time (RSET)

## Abstract

The prevalence of infectious diseases and rapid population expansion has increased the number of medical facilities. Due to the patients' limited mobility, these hospitals are more susceptible to fire disasters. Both Pathfinder and Fire Dynamic simulators were used to calculate the required safe evacuation time (RSET), heat release rate, visibility, temperature, CO, and oxygen effects on temperature, and available safe evacuation time (ASET). The safety egress of the medical hospital was then evaluated by comparing the available safe evacuation time (ASET) and required safe evacuation time (RSET). The simulation findings showed that the egress guides depend on delay time when delay time increases the egress guides drop. We also studied the importance of egress route decisions and suggested that at least 20 to 30 egress guides be on duty in the medical facility. The safety criteria for the medical facility have been proposed based on the delay time with the normalized egress guides. The high oxygen concentration in a hospital can put the medical staff and patients in danger and limit the required safe egress time to less than 150s. The proposed measures can be used to assess the evacuation safety of a typical medical hospital in use relatively quickly and efficiently.

## 1 Introduction

In recent years, evacuation has become a prominent topic. Based on pedestrian age, personality, gender, health status, and many other factors pedestrian behaviors exhibit significant variation. The vast throng will interact strongly on a physical and psychological level. Studies on pedestrian mobility and evacuation are primarily focused on public settings, including schools [1], shopping malls [2,3], and subway stations [4]. There has only been little research on hospital evacuations [5,6], as well as wheelchair evacuations [7,8].

The performance of egress in high-rise buildings is influenced by various factors, including the building's design, the demographics and training of its occupants, the availability of staff, and fire safety installations, among others [9].To quantitatively evaluate available safe evacuation time (ASET) and required safe evacuation time (RSET), Rie et al. [10] used artistic simulation to provide an information transfer function between agents that permits bypass of evacuation by danger as well as the individual setting of the evacuation route and start time for each evacuee. In Hunt et al. [11] the building Exodus model has been used and represents the modeling theory behind an Algorithm presented for both vertical and horizontal evacuation. According to Mufeng Xiao et al. [12] utilizing Pathfinder to apply the physical features of library patrons and the movement speed that reflects these traits, the evacuation time might be decreased. Hung et al. [13] investigated potential fire safety risks and recommendations for small old people rehabilitation facilities for improving fire safety were provided. Many other related types of research are being done using underground complexes for a fire emergency. One of the same research was done by Li et al. [14].

Fire safety guides do not recommend using an elevator during a fire emergency evacuation but Boonngam and Patvichaichod examined pedestrian fire egress scenarios of a multi-story building using a pathfinder. They found that the evacuation efficiency increased by 5.84 % when occupants used the elevator as an evacuation route during a fire scenario [15]. Ronchi et al. [16], Wal et al. [17], and Fu et al. [9] conducted a pedestrian behavioral study on fire during a fire emergency. Abdelghany et al. [18], studied ways to improve pedestrian egress models. Cho et al. [19], and Xie et al. [20], have done similar studies to improve egress models by taking way-finding instructions into account. Numerous kinds of research have been done on pedestrian egress simulation to gauge fire safety [21]. Annunziata al [22]. conducted fire exercises and then carried out egress simulations based on the results he claimed that in case of a fire, the smolder lowers the occupant's extreme speed, and emergency lighting in the ward doesn't provide adequate visibility when there is smoke. Tsungjung Cheng discussed the evacuation route design in a hospital building in Taiwan [23]. But even in many papers, investigations, and findings, hospitals lack proper planning and standardized evacuation procedures. The international fire standards and guidelines need national cooperation [23]. Some researchers used pathfinder to simulate a large population in different facilities. A computer simulation was performed by Maohua Zhong et al. [24] to analyze the exodus of a sizable population from a metro station. Similarly, Jiawen et al. [25] studied fire emergency evacuation from subway stations by using Pathfinder software.

In response to fire and explosion hazards, several safety institutions and public health management authorities have developed recommendations that include directions for the safe handling of oxygen. NFPA and public sources cite a claim that the fire and explosion on April 24, 2021, in an oxygen tank in Baghdad caused at least 82 fatalities and hundreds of injuries, with the number of deaths likely to rise owing to severe burns, the source added [26]. An intensive operation room (OT) in a confined space can result in an uncontrolled environment enriched with oxygen, which constitutes approximately 21 % of the Earth's atmosphere under normal conditions, but when its concentration exceeds 23 %, it can cause fire and explosion hazards [27]. Medical oxygen is not a direct hazard, but it can cause problems if it comes into contact with oils, greases, or fats.

It tends to self-ignite and strongly promotes the combustion of substances (including ointments, gels, and disinfectants). Fire safety analyses have confirmed this, demonstrating that intensive OT and the presence of combustible materials in hospitals facilitate rapid ignition and the spread of fires [28]. Materials that do not burn in air, such as fire-resistant materials, can burn vigorously in oxygen-enriched or pure oxygen-enriched air [29]. Additionally, oxygen considerably raises both the flame temperature and the rate of burning. Electrical devices particularly specialized medical equipment, power cables, extension cords, air conditioners, disinfectants containing alcohol or hydrocarbons, and special medical equipment are examples of igniting devices that can start fires when the air in hospitals is enriched with oxygen [29]. The fact that oxygen is tasteless, odorless, and colorless adds another layer of complexity. The possibility of fire also increases because there are no physiological signs that would immediately notify staff of the presence of an uncontrolled oxygen-enriched atmosphere [30].

The evaluation of egress safety in nursing institutions, where egress guides are crucial due to bad patient conditions, is lacking in the literature even though many studies on fire or egress simulation have been undertaken thus far. Additionally, no studies have been done to increase the egress safety of nursing institutions that are already in use. This study assessed egress safety from a medical hospital using fire and evacuation simulations. (1) The Pyro-sim fire dynamic simulator was used to determine the safe evacuation time (ASET). Volume concentration key variables (temperature, visibility, CO, $O_{2}$, and ${CO}_{2}$) and tenability criteria were analyzed. (2) Using Pathfinder egress simulations were run based on occupant characteristics, and the required safe egress time (RSET) the amount of time needed for all inhabitants to leave the building—was computed. (3) By contrasting the available safe evacuation time (ASET) and required safe evacuation time (RSET), pedestrian evacuation safety was assessed considering evacuation guidelines, and delay time. (4) The normalized egress guidelines and delay time were used to construct the egress safety criteria. (5) Python was used for data analysis.

### 1.1 Research significance

The hospitals are vulnerable to fire incidents because of patients' reduced mobility. Several guides are required to evacuate the patients from the medical facility in case of a fire incident. This study is based on fire and pedestrian egress simulations by considering different delay times and increasing egress guides. Occupants’ safety is proposed by changing the route pattern during evacuation. Overall evacuation delay times and the number of guides are the main variables in this study. Based on simulations, egress safety criteria have been suggested, and these criteria can be used to evaluate the safety of a medical Hospital.

## 2 Fire scenario and simulation model

### 2.1 Building simulation parameters

**Fig. 1:**
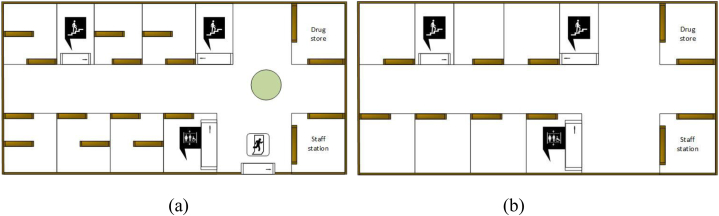
Medical facility layout: (a) Ground floor, (b) First floor.

The prototype medical facility floor plan was selected from a previous experiment carried out by Lei et al. [31]. As illustrated in Fig. 1 and (a) shows the ground floor layout, while Fig. 1(b) depicts the first-floor layout, the wall's height is 2.75m and a floor size of 1173 m^2^ chosen. Fang et al. [32] performed experiments and models of exit-selection behaviors during a building evacuation. The findings revealed that staircases were the primary mode of high-rise building escape. Many researchers have researched stairwell safety [33]. The Code of Design on Building Fire Protection and Prevention(GB50016 – 2006) states that four possible configurations of staircases located in various areas of a building can be taken into consideration [31].In this investigation, we used scenario 2 from a prior study by Lei et al. [31] Which had two wide stairs on either side of the building and one 4m-wide center exit. Several studies have also been conducted to investigate the fire performance of exterior wall cladding using the British Standard BS 8414-1 [34] as an investigative test method. The test apparatus must have a main wall and a wind wall, as specified in BS 8414-1.

### 2.2 Evacuation safety assessment procedure

The evacuation safety was evaluated in this study by comparing the required safe evacuation time (RSET) and available safe evacuation time (ASET). A fire dynamic simulator is used for the required safe evacuation time for all patients, and Pathfinder is used to address egress simulations [35]. Pathfinder is an agent-based model which is used to predict behaviors based on occupant characteristics. A fire dynamic simulator developed by NIST is used to determine visibility, carbon monoxide, oxygen, and carbon dioxide. When the values of these gases exceed the suggested deadline is very harmful to occupants’ health and limits the evacuation during a fire disaster. Below Table 1 reports the critical value limit criteria proposed by the National Fire Protection Association (NFA) [36]. The Pyro-sim analysis results were used in this study to compute the times necessary for visibility, CO, ${CO}_{2}$, $O_{2}$, and temperature to meet each tenability criterion.

**Table 1:** Reported tenability limit for 5-min exposure to common gases [37].

Physical criteria	Gas species	Incapacitation	Criteria
	CO	6000–8000 ppm	Less than 1400 ppm
Tolerance tenability	$O_{2}$	10–13 %	>15 %
	${\text{CO}}_{2}$	7–8%	<5 %
Visibility tenability			5m
Temperature (°C)	°C	**Reported tolerance time(sec)**	
	126	420	
	180	240	
	205	180	
Temperature tenability			>60°

### 2.3 Fire simulation model

**Fig. 2:**
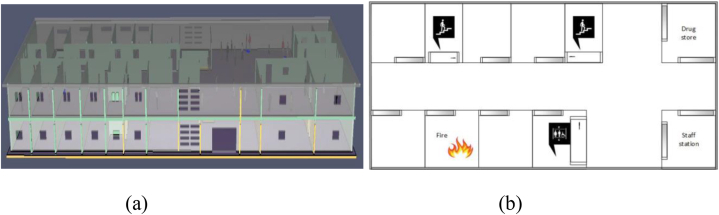
Medical facility model: (a) Pyro-Sim simulation model, (b) Location of fire combustion and egress routes.

In this simulation Fire Dynamic simulator, the 2019 version was used [user guide], Fig. 2(a) illustrates the FDS model used for the fire dynamic simulation, and Fig. 2(b) shows the burner location and pedestrian evacuation routes. An elevator and two staircases (STA, STB) were built as egress routes. It was assumed that the second floor of the prototype medical hospital was under fire due to an unknown reason. We have used short-form STA and STB for our convenience in this study. In a fire emergency, using elevators to leave the building is typically prohibited. However, some countries permit the use of elevators in emergencies but under wholly distinct operations. Medical facilities use beds, and wheelchairs (self or aided) to evacuate the patients. The fire simulation was run for 600s with a 20°C beginning temperature assumption. The input features of the FDS model are listed in Table 2.

**Table 2:** Input fire characteristics.

Input		Properties
Total heat release rate (HRR)		29,854.0 kW
Simulation time		600s
Burning material	Fuel type	Polyurethane foams (GM29)
	Formula	CH_1.8_
	Co yield $\left(\;Y_{co}\right)$	0.042
	Soot yield $(Y_{S}$)	0.228
Air species	Measuring devices	Visibility(m)
		CO(ppm)
		${CO}_{2}$ (%)
		$O_{2}$ (%)
Compartments	Fire growth rate	Medium (0.01172 kW/m^2^)
	Burning area	1m × 1m
	bed	4665
	wardrobe	270
Max HRR	chair	454
	curtain	240
	Refrigerator	2125
	TV	239

The mesh size and D for fire simulation for this simulation are based on guidelines preferred by the FDS user guide.

1 $$D^{*}={\left(\frac{Q^{\cdot }}{\rho_{h}T_{h}C_{p}\sqrt{g}}\right)}^{\frac{2}{5}}$$

where $\dot{Q}$ = The total heat release rate in kW,

$\rho_{h}=Air\;density$,

$T_{h}=Room\;temperature\;at\;initial\;state$,

$C_{p}=Air\;specific\;heat$,

g = Gravitational acceleration respectively.

**Fig. 3:**
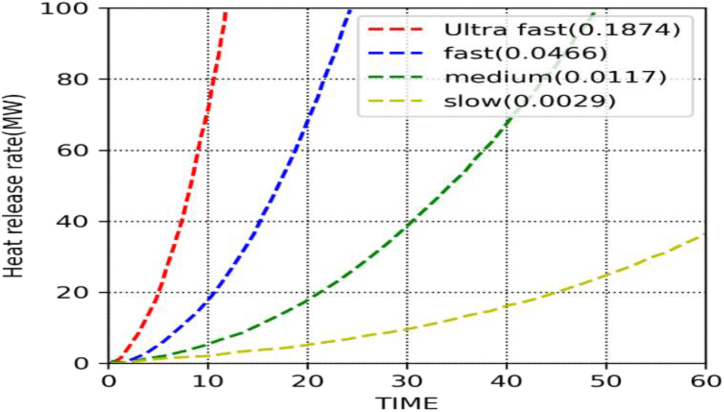
Fire ramp t^2^ curves.

By using equation (1) mesh size was set at (0.25 × 0.25 × 0.28) m. Where, Q is the overall heat release rate in kW, the specific heat $C_{p}$ is 1.005 kJ/kg-K, the ambient temperature (T) is 293 K, and the acceleration (g) caused by gravity is 9.8 m/s^2^. The fuel chemical characteristics are chosen from the values listed in the Society of Fire Protection Engineers handbook [38]. The National Center for Forensic Science's database was used to determine the heat release rate of each combustible [39], which was presumed to be beds, tables, and chairs. Equation (2) was used to model the t-squared fire curves depicted in Fig. 3 by calculating the heat release rate (Q) as a function of time:

2 $$Q={\alpha t}^{2}\left(kW\right)$$

Where Q is the total heat release is the time, and α is the fire growth coefficient. The classification of α normally includes "slow," "medium," "rapid," and "ultrafast." The "Structural Design for Fire Safety" was used as the basis for the medium level (α=0.0117) that was used in this investigation [4]. The smoke exhaust system and fire extinguisher were assumed to be off for the fire simulation, and the burner area was 1 m^2^.

## 3 Simulation

### 3.1 Tenability limits

To evaluate life safety hazards using a numerical simulation tool, quantitative tenability criteria are required. An average individual exposed to an extreme temperature for more than a few minutes is likely to get burned and die. Table 1 reported the tolerance time values concerning temperature and also for other toxic gases.

**Fig. 4:**
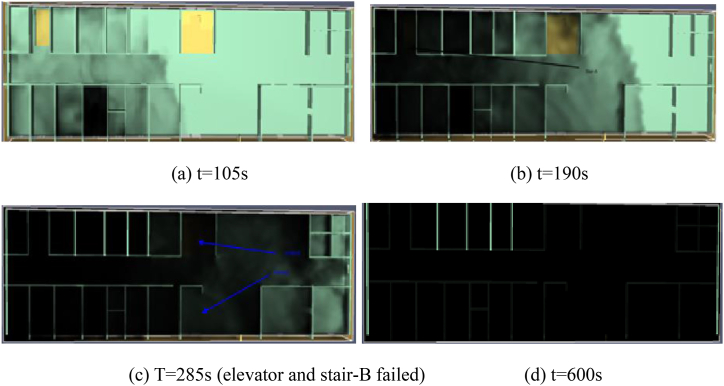
Simulation of smoke behavior at different time intervals.

**Fig. 5:**
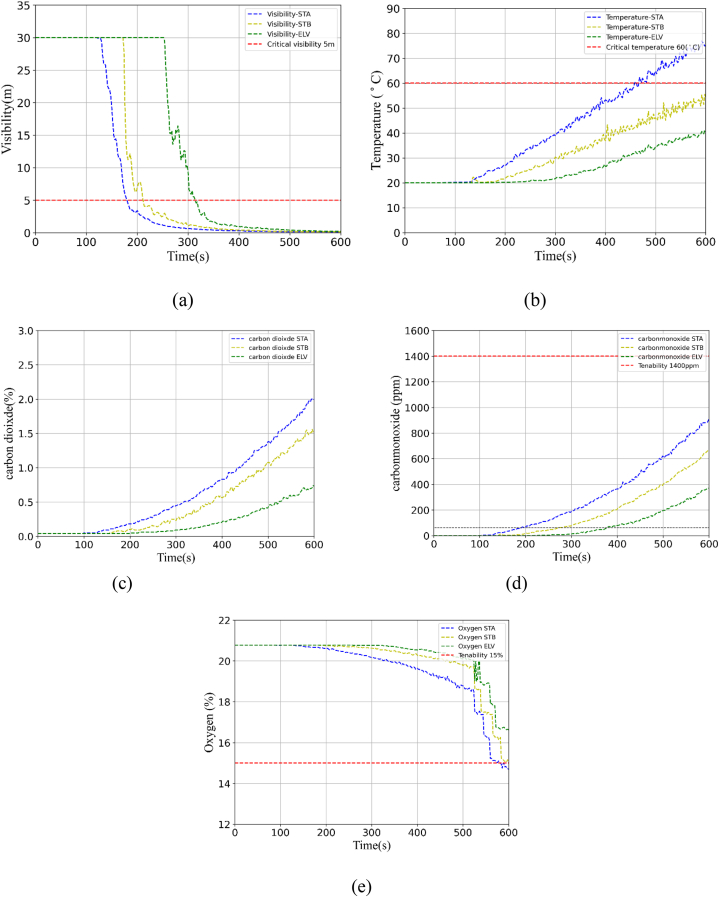
Simulation results: (a) visibility; (b) Temperature; (c) Carbon dioxide (%); (d) Carbon monoxide(ppm); (e) Oxygen (%).

Fig. 4(a)–(d) shows the smoke behavior on the second floor of the hospital building. When the fire first started during time t it began to spread across the room as shown in Fig. 4(a). After about 105s it covers the whole room and fills in the hallway running towards stairs (STA and STB), and the elevator. After 190 s, smoke covered the stairwell (STA) and began moving towards stairwell STB, as shown in Fig. 4(b). For approximately 210 s, smoke covered STB, and by 285 s, it had reached the elevator area, as depicted in Fig. 4(c). Fig. 4(d) illustrates that by 600 s, the entire floor was engulfed in smoke. Fig. 5(a)–(e) shows the simulation results for each of the three egress routes (STA, STB, and the elevator) for the fire duration (t) in terms of temperature, visibility, $O_{2}$, CO, and ${CO}_{2}$. These simulation results were carried out at normal oxygen levels. The tenability criteria are shown by the black highlighted line in each graph and are considered available safe egress time for each factor. Table 1 lists the tenability criteria for each species. The visibility criteria for STA (194s), STB (215s), and an elevator(310s) are shown in Fig. 5(a). The temperature at STA was highest above 70° and achieved tenability criteria but the temperature for STB and elevator were 54°, and 40° shown in Fig. 5(b). The CO, and, ${CO}_{2}$ modeling results are shown in Fig. 5(c) and 5(d) respectively. The highest CO and ${CO}_{2}$ concentration was achieved in STA. The simulation results for $O_{2}$ is shown in Fig. 5(e), respectively. The tenability criteria were attained by STA and STB. All species were not met the tenability criteria, based on assumed fire duration(t). The visibility mostly influences the safe egress time of the occupant. That's why we set 192s as the acquired safe egress time for occupants in this prototype medical hospital.

**Fig. 6:**
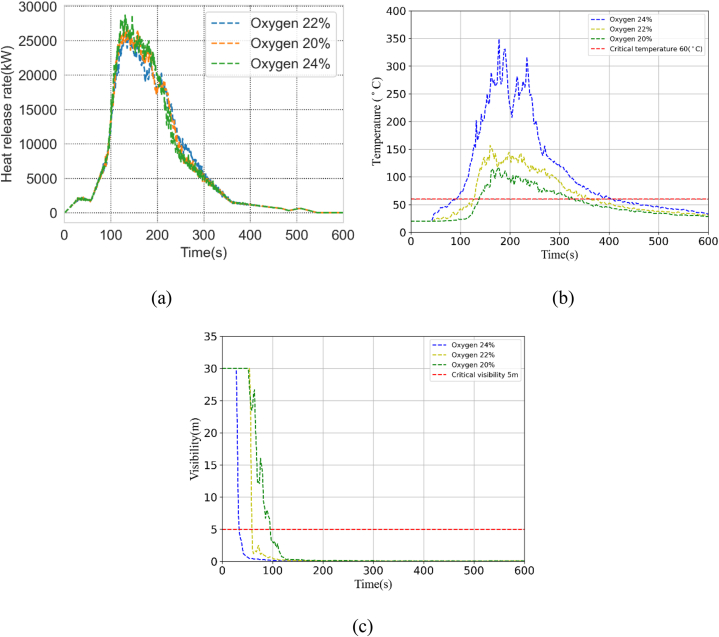
Impact of Oxygen Concentration on (a) Heat release rate, (b) Temperature, (c) Visibility.

Another oxygen concentration simulation was performed in the corridor which is located in front of the fire incident room. In the above simulation, the oxygen concentration was within the normal range but for the below simulation initial oxygen concentration varies from 21 % to 25 %. Oxygen concentration impacts temperature, visibility, and other quantities, as shown in Fig. 6(a),(b), and 6(c). Fig. 6(a) illustrates the heat release rate's effect on different oxygen concentration levels. Due to high oxygen levels, tenability thresholds can be reached within seconds inside the fire room, as shown in Fig. 6(b). Fig. 6(c) demonstrates that visibility drastically decreases at high oxygen levels, which could be dangerous for patients trying to evacuate if not properly prepared. The period available for medical staff to conduct self-evacuations and other forms of evacuation is directly impacted by the dynamics of the fire growth. It is anticipated that, under typical hospital room conditions, a fire would start and spread at a normal rate, taking 5 min to reach flashover. However, this period would be drastically shortened if exposed to an atmosphere high in oxygen. The calculated available safe evacuation time (ASET) time, which is related to the occurrence of human-critical circumstances (high temperature, pungent smoke), is even less than the authorized duration of 5 min.

According to the simulation analysis, the medical staff only have 150s in an oxygen-rich environment to egress patients and themselves. This is a relatively very small available safe evacuation time (ASET) and it is not easy because of fire pre-detection time delays (fire alarm delays). Some serious factors also need to be considered. The visibility in the corridor was considered. The results showed that due to extreme oxygen concentration, the time required to cross the corridor was limited to 50s at 25 % oxygen concentration and declined evacuation drastically. Thus, it indicated that the oxygen concentration can cause serious risk for medical staff in case of a fire incident.

## 4 Occupant egress simulation model

### 4.1 Simulation model

**Fig. 7:**
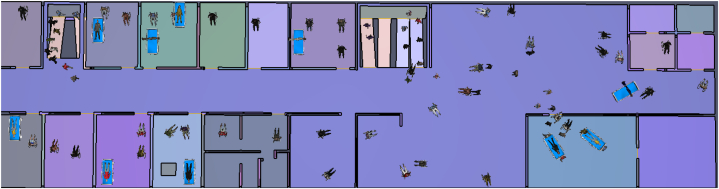
The Pathfinder sampled pedestrian evacuation model.

The sampled Pathfinder model for pedestrian evacuation is shown in Fig. 7. In this simulation, the total number of evacuees and their profile parameters such as evacuee's velocity, evacuee width, and pre-movement time were considered. South Korean Administration Rule for Senior Citizens Act suggests a minimum of 23.6 m^2^ for each patient [35]. In this simulation, we included 110 patients and divided them into their health statuses such as severe, medium, low normal, etc. According to the patient's health status, we categorized the patients into different groups and egress route choices as shown in Table 3. During a fire incident, 12 patients were bed-bound,30 patients were in a wheelchair with assistance,20 patients were in a wheelchair (without assistance),30 were in guided wheelchairs (with assistance), and 40 were output patients. However, some were seriously ill so they were evacuated with their beds with the assistance of egress guides.

**Table 3:** Evacuation type and the number of patients.

Evacuation type	Health status	Behaviors	Number of patients	Ratio (%)
Bed bound	Severe	Wait for assistance	12	9.8
Wheelchair (guider)	High	Wait for assistance	30	24.5
Wheelchair (self)	Medium	-Go to the exit	20	16.4
Output patient	Below	-Go to the exit	40	32.8
Visitors	Healthy	-Go to the exit	20	16.4
**Total**			122	100

### 4.2 Simulation settings

The evacuation characteristics of patients and hospital staff are chosen by considering the real scenarios of the hospital as well as the type of profiles and their speed. The evacuation type and the total number of patients for each profile are listed in Table 3.

Critically ill patients are transferred using hospital beds that are supported by an evacuation assistance team. These hospital beds can be categorized into different types, as outlined in Table 4.

**Table 4:** Bed type for seriously ill patients.

Bed type	Bed size(m)	Assistance needed(per/bed)
Young	0.5 × 0.9 × 0.88	1
Adults Bed	0.9 × 2 × 1	2

The patient and egress guide characteristics and profiles are listed in Table 5. Lee et al. [3] based on egress type the speed, patient's width, and egress guides were determined. The hospital pedestrian egress rules and regulations suggest at least 20 s are required as pre-evacuation time [40]. One doctor,4 nurses,2 physical therapists,2 helpers, and more than 60 caretakers were needed to serve 120 patients. These members of staff were imagined to be egress guides for patients during the simulations. Staff in nursing hospitals often work in shifts, with fewer people working night shifts than day hours. As a result, in this study, hospital staff were assumed as variables during egress simulations. For the egress simulation model, there were between 10 and 80 workers, spaced every 10. It was expected that half of the workers in each case were listed in Table 6.

**Table 5:** Profile parameters.

	Profile	Mobility feature	Count	Area covered	Speed(m/s)	Preparation time(s)
	Doctors	Move without assistance	1–3	0.402	1.10-1.50	
	Nurse	Move without assistance	2–6	0.357	1.10-1.50	
**Hospital staff**	Emergency team	Move without assistance	5–50	0.403	1.10-1.50	
	Others/firefighters	Move without assistance	2–5	0.358	1.10-1.50	
	Bed	Need of assistance		0.723	0.34-0.50	20–25
**Visitors**	Walk			0.402	1.0–1.4	
**Patients**	Wheelchair (guider)	Need of assistance		0.71	1.3	10–15
	Wheelchair (self)			0.7	1.4	10–15
	Walk			0.49	0.6

**Table 6:** The number of hospital staff.

	Doctors	Nurses	Physiotherapists	General worker	Total guides
**1**	1	2	1	1	5
**2**	2	1	1	7	10
**3**	2	2	1	10	15
**4**	2	3	1	14	20
**5**	2	2	2	14	25
**6**	2	2	1	25	30
**7**	2	4	2	27	35
**8**	1	3	1	36	40
**9**	1	4	2	38	45
**10**	2	5	1	42	50

During a fire, the delay time is the period it takes for a patient and egress guide to begin exiting. The delay period is the sum of a fire detection time, fire notification time, and pedestrian pre-movement time. The delay can be minimized by promptly considering notifications or warning signals or by closely monitoring closed circuitry cameras (CCTVs). The egress delay time, however, increases if there is a lack of personnel who are qualified to manage fire emergencies, CCTVs, and broadcasting facilities, raising the RSET.

**Fig. 8:**
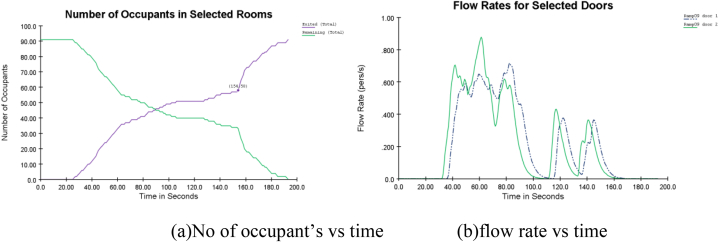
Occupants evacuation time and flow rate (Egress guides = 20).

**Fig. 9:**
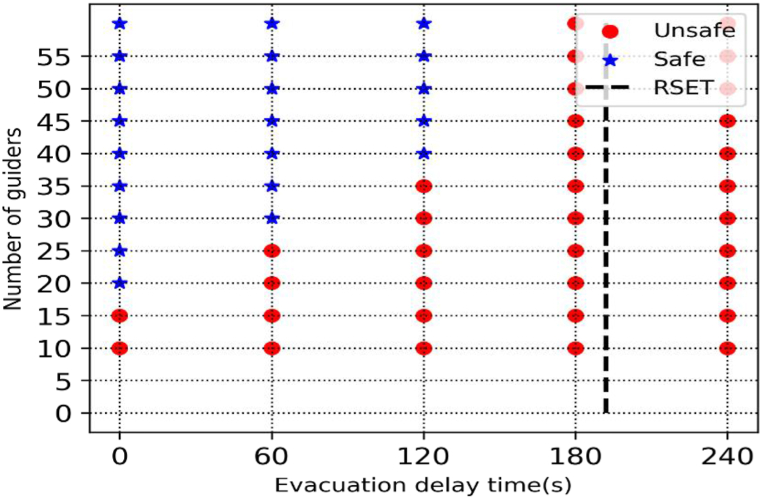
Evacuation delay(s).

In this study, the evacuation safety time was assessed in terms of delay time by running simulations with the delay time set to 0–360s by an interval of 60s. Table 7 presents the time delay and the patient egress times with various random delays. Fig. 8, Fig. 9 shows the number of occupants inside the selected rooms concerning evacuation time. Whereas Fig. 8(b) shows the flow rate concerning time. The number of occupants and their evacuation time are shown in Fig. 8 (selecting egress guides = 20) (see Fig. 9).

**Table 7:** shows the time delay variables and egress time.

Guides	Random route allocation (delay time(s))					
0	0	60	120	180	240	300
**10**	407	467	527	587	647	707
**15**	342	407	467	527	587	647
**20**	293	336	396	456	516	576
**25**	242	315	375	435	495	555
**30**	241	290	350	410	470	530
**35**	171	276	336	396	456	516
**40**	168	233	293	353	413	473
**45**	159	226	286	346	406	466
**50**	162	218	278	338	398	458
**55**	161	215	275	335	395	455
**60**	149	205	265	325	385	445

### 4.3 Simulation results

The required safe evacuation time (RSET) for each case was determined to be the moment when everyone had safely evacuated the building. Table 8 shortlists the number of evacuees who failed to egress during available egress time (ASET). When the egress time was set at 192s which was the lowest visibility attained during fire simulations. About 22 persons failed to exit the building when there were 10 egress guides present and a delay of 0 s. When the number of numbers of egress guides was 10 at a delay time was 0 s around 22 people failed to egress the building. When the egress guide's number reached 20 the evacuees safely evacuated the building. When the delay time is maintained and increased the egress guides everybody egressed safely. When the delay time increased above 192 s everybody failed to egress. When the number of egress guides increases, the number of egress evacuees tends to rise as well.

**Table 8:** Number of people who failed to egress.

Number of Egress guides	Delay time(s)				
	0	60	120	180	240
**10**	22	29	34	40	47
**15**	48	31	37	47	52
**20**	0	10	39	47	56
**25**	0	6	40	55	60
**30**	0	0	18	54	66
**35**	0	0	15	55	71
**40**	0	0	0	25	74
**45**	0	0	0	28	80
**50**	0	0	0	34	84
**55**	0	0	0	30	88
**60**	0	0	0	32	89

The British Standard Institute defined the egress delay for public safety [40] as shown in Table 9. The delay times are categorized as P1(<180s), P2(300s), and P3(>480s) based on the professional skills of the fire safety team and the staff during a fire event and also the accessibility of closed-circuit cameras (CCTVs) and messaging devices. When designing for life safety the ASET> $\Delta t_{esc}$ where $\Delta t_{esc}=\Delta t_{a}$ + $\Delta_{tevac}$.

**Table 9:** The egress delay for public safety.

Facility type	P1	P2	P3
Medical facilities, elderly nursing homes, residential areas (A large number of evacuees need assistance)	Less than 180	300	Greater than 480

P1: Voice communication or live; P2: Visual warning and using trained staff; P3: Fire warning system, and staff without relevant training.

$\Delta t_{esc}$ is the egress time to reach a place of safety;

$\Delta t_{evac}$ is the required time after alarm for evacuees to egress to a safe place;

$\Delta t_{a}$ = The time of ignition of the alarm.

**Fig. 10:**
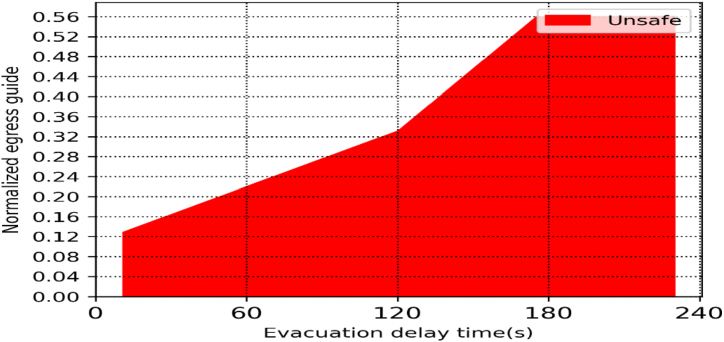
Normalized evacuation guides.

The required safe evacuation time (RSET) is often chosen based on the occupancy type's egress safety time during performance-based egress safety design. The number of egress guides and the delay time should be considered when evaluating the medical hospital's egress safety, according to data from prior studies. In general, the number of evacuees in a hospital can be estimated based on the size of the building, the number of patients, the number of staff, and the number of caretakers. As a result, the normalized number of egress guides is an objective statistic that can be used to evaluate the egress safety criteria because it considers both the area and capacity of the facility as well as the number of patients. The pedestrian evacuation safety criteria are shown in Fig. 10. Using the average number of egress guides and the delay time at a typical prototype medical facility, one can determine whether an evacuation was successful or unsuccessful. The left space indicates successful egress, whereas the red region indicates failure. These suggested criteria can be used to assess egress safety and set up supplemental measures in typical nursing institutions that are currently in operation. In other words, using proposed criteria based on the number of egress guides and delay time computed by accounting for the broadcasting facilities, CCTVs, and staff competency in handling fire situations, it is possible to determine whether the egress safety in a functioning medical hospital is safe or unsafe.

There are two ways to improve egress safety when it has been deemed dangerous. The most important factor is a delay, delay time should be decreased by improving staff expertise in responding to a fire. By installing closed circuitry cameras (CCTVs), fire detection systems, or using building broadcasting equipment, the evacuation can be smooth and successful. To assure safe egress or shorten the delay time, the number of egress guides needed can be determined using the provided criterion. Medical facilities can improve egress safety while lowering costs and boosting guiders' skills as well as patient handling equipment for example beds, chairs, wheelchairs, etc.

## 5 Discussion

Hospital evacuation protocols are essential elements of disaster preparedness and must be meticulously designed to guarantee the safety and well-being of patients, staff, and visitors during emergencies [41]. Considering the unique challenges present in a hospital environment, such as patients with limited mobility or special medical needs, a comprehensive and personalized approach to evacuation is required. The most important finding from this study is the recognition of the significance of available safe evacuation time (ASET) and it is affected by other factors such as oxygen levels, and rescuers as guides during evacuation scenarios. Our initial findings revealed the potential risk associated with high concentrated oxygen levels and its impact on pedestrian evacuation. The results demonstrated that the visibility reached its critical level in less than 100 s. On the other hand, when oxygen levels were normal, the available safe evacuation time (ASET) was considered to be 192 s. The temperature tenability was 500 s for the normal oxygen scenario, but it decreased to approximately 150 s when oxygen levels were high. These findings highlight the impact that high concentrated oxygen levels can have on the available safe evacuation time (ASET).

This study evaluated safety time in terms of delay time by conducting simulations with a range of delay times from 0 to 360 s, incremented by 60 s, under normal oxygen concentration levels. The delay time for patients occurs when they briefly wait in designated rooms or areas designated for rescue assistance, awaiting the arrival of rescuers. Multiple examples can be found in the literature, including the significance of incorporating refuge floors in Hong Kong's legal framework [42]. According to Table 8, the number the patients who experienced failure due to time delays involving rescuers can be found. Studies have revealed that ensuring patient safety and creating a safe environment for patients and staff during hospital emergency evacuations are critical and necessary factors [43,44]. A study found a low-moderate level of safety in Iranian hospitals during disasters [45].In addition, the findings of a review study revealed a modest level of preparedness for the emergency department of Iranian hospitals in disasters [46]. This current study analyzes the delays in preparedness to demonstrate how much delays can afford this prototype model hospital. Given the importance of patient and hospital safety during the evacuation process, it is advised that health managers increase hospital safety and preparation.

Many criteria must be considered to ensure the safe evacuation of all patients at a hospital. Communication, command and control, patient evacuation, and critical care are just a few examples. According to the findings of this study, the number of rescuers plays a vital influence in health facility evacuation and the time required to reach the refuge area. Rahouti et al. [47] demonstrate that when the number of attendants is reduced, overall evacuation time increases. This study addresses the importance of having egress guides on duty at all hours of the day and night duty to achieve complete evacuation before tenability criteria. Previous fires in hospitals such as the Royal Marsden(complete evacuation), Great Ormond Street(partial evacuation), North London Forensic Service (full evacuation), and Northwick Park Hospital (partial evacuation) [48]. Some of these hospitals (guides) did an excellent job, evacuating the whole hospital population in 28 min. This is because of the large number of guides and staff training. The findings of our research have significant implications for enhancing hospital evacuation protocol and preparedness and highlight the need for training programs for the staff in efficiently deploying resources during evacuations.

## 6 Conclusions

The following results were achieved after conducting simulations of a model medical hospital's fire and evacuation.

Using FDS, visibility, CO, ${CO}_{2}$, oxygen, and temperature of a typical prototype medical hospital were examined based on the length of the fire. The tenability of the criteria was computed by taking the lowest value as available safe evacuation time (ASET) and comparing the results for other egress routes. The reduction in visibility brought on by the spread of smoke served as the primary basis for determining the available safe egress time (ASET). Prototype medical hospital evacuation simulations were run using Pathfinder software by taking the egress characteristics such as the number of egress guides and delay time(s) of the evacuees. According to the simulation's findings, the required safe evacuation time (RSET) increased as the delay time and egress guide number decreased, and at least 30 egress guides should be on duty to tackle any emergency for this prototype hospital and can adjust 60s delay during a fire incident.

Fires in a highly concentrated environment can spread quickly and can reduce the response to the fire as well as the evacuation time. This study suggests the separation of critical health care wards from normal hospital wards. By contrasting the available safe evacuation time (ASET), and required safe evacuation time (RSET), the evacuation life safety of the prototype medical facility was assessed. The normalized number of evacuation guides was proposed based on delay time simulation findings. The suggested egress criteria can be used for a typical medical hospital to evacuate occupants quickly and efficiently.

The study only gives an insight into the medical facility without a smoke exhaust system. The egress safety system with smoke exhaust would greatly impact the evacuation and needs to be studied in the future.

## Data availability statement

The data will be provided on the request.

## CRediT authorship contribution statement

**Mohsin Ali Shaikh:** Software. **Rehmat Karim:** Methodology, Conceptualization. **Nashiru Mumuni Daniel:** Writing – review & editing, Software. **Mujeeb Ali Khan:** Software.

## Declaration of competing interest

The authors declare that they have no known competing financial interests or personal relationships that could have appeared to influence the work reported in this paper.
